# Blood biomarkers differentiating viral versus bacterial pneumonia aetiology: a literature review

**DOI:** 10.1186/s13052-020-0770-3

**Published:** 2020-01-09

**Authors:** Jithin Thomas, Aiste Pociute, Rimantas Kevalas, Mantas Malinauskas, Lina Jankauskaite

**Affiliations:** 1 University Hospital of Southampton, Southampton, Hampshire, UK; 2 Medical Academy, Lithuanian University of Health Sciences, Kaunas, Lithuania; 3 Medical Academy, Hospital of Lithuanian University of Health Sciences, Kaunas, Lithuania; 4 Institute of Physiology and Pharmacology, Lithuanian University of Health Sciences, Kaunas, Lithuania

**Keywords:** Community-acquired pneumonia, CAP, viral pneumonia, Virus-induced pneumonia, Bacterial pneumonia, Biomarker, Marker, protein, Interleukin, Chemokine

## Abstract

**Background and objectives:**

The goal of this literature review is to compare current studies regarding the accuracy of different serum markers in differentiating viral from bacterial pneumonia in the pediatric population with what is employed in the medical settings at present. Currently there is still a lack of significant research, that would give us evaluation on biomarkers benefits towards getting a definite diagnosis of pneumonia. Finding out the potential of biomarkers to differentiate between viral and bacterial pneumonia is also important because knowing the exact pathogen would prevent irrational use of antibiotics. At present, irrational, broad-spectrum antibiotic use and increasing antibiotic resistance in microorganisms are still one of the greatest challenges in clinical settings. The use of biomarkers in clinical practice would not only facilitate accurate diagnosis, but would also help to reduce the amount of antibiotics overuse.

**Materials and methods:**

Literature search conducted on Medline and Google Scholar using a combination of terms. Articles that were in English and within ten years of the search date were manually sorted according to inclusion and exclusion criteria. Results: Initial search returned *n* = 13,408. After activating filters, *n* = 140 were identified of which *n* = 12 included for literature review.

**Conclusions:**

Rise or drop in the concentration of a single marker is not accurate enough for predicting viral/bacterial community acquired pneumonia. This is because there is overlapping to a varying extent depending on the marker cut-off values, detection methods, analyses, the desired specificity, and sensitivity. Furthermore, the presence of mixed infection makes almost all markers suboptimal to be used universally. New markers such as MxA1 and HMGB1 gave promising results. However, to replicate a similar testing condition in a clinical environment may not be practical. Another approach is to make use of more than one marker and combine with clinical signs and symptoms. This may not be cost-effective in many clinical settings; nevertheless, in many studies, marker combination greatly improved the predictive power.

## Introduction

Community-acquired pneumonia (CAP) is estimated to cause 31.1 per 100,000 deaths globally in the population under the age of 19 [1]. According to the epidemiological data, approximately 152 million cases of CAP are diagnosed every year in children under the age of five worldwide, of which, approximately 10–20 million are severe cases requiring in-patient treatment [2]. However, there has been a drop in the incidence and mortality of CAP with the introduction of vaccination against *Streptococcus pneumoniae* and *Haemophilus influenzae* [3–5]. Thus, viral pathogens have become significant in causing CAP. It is estimated that approximately 50–70% of cases of CAP are viral-induced in children under the age of 5 [6]. However, antimicrobial drug use remains one of the biggest challenges in viral CAP cases [7], especially in children. In addition, diagnostic limitations in differentiating viral and bacterial pathogen in CAP causes increased antibiotic use and contributes to antibiotic resistence growth [8].

The biggest challenge remains to differentiate common respiratory viral pathogens from bacterial causes. Clinical signs and symptoms of CAP of viral and bacterial origin overlap significantly [9]. The uncertainty is further exacerbated by the fact that direct isolation of possible causative agent from the lower respiratory tract is invasive and therefore rarely performed [8].

Consequently, indirect methods are utilized to isolate the organism. These include polymerase chain reaction (PCR) of throat swab, gram stain, and culture of nasopharyngeal aspirate, and blood cultures. However, interpretation can be difficult as children are found to be asymptomatic carriers of a range of organisms and a positive result on PCR may not be indicative of the cause of CAP [8]. C-reactive protein (CRP), and White blood cell count (WBC) are often part of the diagnostic workup in an inpatient setting. However, the changes observed are not specific to predict causative pathogen.

Instrumental diagnostics, such as a chest X-ray is not sensitive or specific and is not recommended in the initial diagnosis of a suspected CAP [10]. Radiographic changes which show patchy bilateral involvement may suggest a viral aetiology, however, this is not specific [10].

A great deal of attention, therefore, is given to quantitative changes in different serum markers to make better conclusions. Owning to the difference in the immunological and inflammatory response induced by bacteria and viruses, the disparity in the levels of specific markers may give an objective value that may equip us with better prediction power regarding aetiology. Many research studies have explored the different serum markers, but the conclusions are conflicting [7, 11–13]. Therefore, an intuitional review is vital to provide enough clarity to bridge the scientific gap. The underlying principle of this research is to summarise literature analysing different biomarkers and provide an overview. The use of new biomarkers in clinical practice would not only facilitate accurate diagnosis, but would also help to reduce the amount of antibiotics used.

## Materials and methods

### Eligibility criteria

Studies that included pediatric patients with the diagnosis of CAP focusing on specific new diagnostic markers of viral and/or bacterial pathogen and not older than 10 years were eligible to be included in this review (Table 1).

**Table 1:** Filters that were applied after the initial search

Date range: Within ten years (until 1st of July, 2019)	
Species: Human	
Language: English	
Age: Birth-18 Years	

### Inclusion and exclusion criteria

We excluded all studies focused on adult CAP. Additionally, all studies analyzing pediatric patients with comorbidities and diagnosed with CAP were excluded from our review. Studies which were not restricted to pneumonia, or selected CAP other than viral/bacterial were excluded as well. We also excluded animal studies, literature review, systematic review and metanalysis. All inclusion and exclusion criteria are represented in Table 2.

**Table 2:** Criteria for inclusion and exclusion

Inclusion	Exclusion
Free text (also an external link to other sites)	Adult
Viral and bacterial aetiologies	Pneumonia with other aetiologies than viral/bacterial (human immunodeficiency virus, immunodeficiency, cancer, post-transplantation)
No concomitant diseases	Literature reviews
Age 0–18 years	Metanalysis
	Systematic reviews
	Abstracts only with no external link

### Search strategy

Literature was identified by two independent reviewers searching Medline and Google Scholar until the 1st of July 2019. The search strategy included a combination of the following terms: “Community-acquired pneumonia” OR “CAP” OR “viral pneumonia” OR “virus-induced pneumonia” OR “bacterial pneumonia” AND “biomarker” OR “marker” OR “protein” OR “interleukin” OR “chemokine”.

### Data extraction

After all the primary studies were collected, all the study characteristics were extracted as follows: the author of the study, date of enrolment, where the study was conducted, patient age, type of the study, and target biomarker. Numerical data extracted included the number of CAP patients in each study and number of viral and bacterial cases. Moreover, the main findings were summarized in conclusion and notes (Table 3).

**Table 3:** Critical summary of the sixteen articles that were filtered after the initial search

Author (REF)	Date	Country	Age range	Type of the study	No. of CAP cases	No. of viral and bacterial cases	Markers tested	Conclusion and notes
Bhuiyan et al. [14]	2019	Australia	≤17 years	Prospective cohort	*n* = 230	Bacterial *n* = 30	CRP, WBC, absolute neutrophil count (ANC)	CRP, WBC, and ANC were higher in definite bacterial pneumonia. The median blood CRP concentration was more than 6 times higher in definite bacterial cases than viral.
						Viral *n* = 118		
						Other *n* = 82		
Huang et al. [15]	2019	China	1 to 13 years old	Prospective cohort	*n* = 40	HAdV pneumonia *n* = 20	miRNAs	miRNAs biomarkers for HAdV pneumonia, at least in the cohort experiment. Importantly, neither pair of miRNAs could independently distinguish HAdV-infected patients from healthy children, highlighting the requirement for combining the two miRNA pairs identified in the present study.
						Healthy n = 20		
Yang et al. [16]	2018	China	0.8 to 9.6 years	Retroperspective cohort	*n* = 321	Healthy controls *n* = 50	YKL-40, IL-6, IL-10, TNF-α, CRP	No significant difference between the levels of YKL-40 in the serum in all 3 pneumonia subgroups. The levels of YKL-40 in the BALF specimens of patients with bacterial pneumonia were significantly higher than with viral pneumonia. The levels of IL-6, TNF-α, and C-reactive protein were positively correlated with the serum levels of YKL-40. IL-10 levels were negatively correlated with YKL-40 levels in all pneumonia subgroups.
						Viral = 104		
						Bacterial = 110		
						Co-infectio*n* = 66		
Wallihan et al. [17]	2018	United States (Ohio)	2 months to 18 years old	Prospective cohort	*n* = 152	Healthy controls *n* = 39	WBC, CRP, PCT	CRP, and PCT values were highter in patients with pyogenic bacteria than those with viral pneumonia or M. pneumonia.
						Pyogenic bacteria = 16		
						M.pneumoniae = 41		
						Viral *n* = 78		
						Undetermined n = 14		
Yang et al. [18]	2018	China	≤10 years	Prospective cohort	n = 82	Healthy controls = 21	HPR	HPR is higher in the mycoplasma pneumonia and the viral pneumonia than in the bacterial pneumonia.
						Bacterial = 21		
						M.pneumoniae = 21		HPR is a potential biomarker differentiate bacterial pneumonia and non-bacterial pneumonia.
						Viral = 19		
Higdon et al. [19]	2017	Bangladesh, Gambia, Kenya, Mali, South Africa, Thailand, Zambia	<5 years	Case-control study	*n* = 3981	HIV-negative tested controls *n* = 601	CRP	CRP was highter with bacterial pneumonia and negatively associated with RSV pneumonia. CRP may be useful for distinguishing bacterial from RSV-associated pneumonia, although its role in discriminating against other respiratory viral pneumonia needs further study.
						HIV-negative with comfirmed bacterial pneumonia *n* = 119		
						HIV-negative with comfirmed viral pneumonia *n* = 556		
Esposito et al. [20]	2016	Italy	< 14 years old	Prospective cohort	110 radiologically confirmed CAP	n = 20 no aetiology identified	Lcn2, SYN4, CRP, WBC	Lcn2 and SYN4 cannot predict aetiology. CRP together with WBC and clinical data, when combined, is the best predictor.
						Bacterial *n* = 74;		
						Viral *n* = 16;		
						Confirmed with PCR from nasopharyngeal swab positive		
Valim et al. [21]	2016	Mozambique	< 10 years	Prospective cohort	*n* = 117	Bacterial n = 23;	Fifty-six markers in a multiplex immunoassay. Hap, TNF receptor 2 or IL-10. The full list of markers was beyond the scope of this review.	Combination of three proteins (Hap), TNF receptor 2 or IL-10, and tissue inhibitor of metalloproteinases 1 provided the best tool to differentiate bacteria from the virus.
						Viral n = 30;		
						Bacteria isolated from blood or pleural fluid;		
						Viruses identified with PCR.		
Esposito et al. [22]	2016	Italy	4 months-14 years	Prospective cohort Multicentre	433 radiologically confirmed CAP	Bacterial *n* = 235;	CRP and PCT, MR-proANP, MR-proADM, WBC, neutrophil percentages	CRP and PCT are better at predicting viral and bacterial aetiology than others.
						One or more viruses *n* = 111;		
						Unknown *n* = 87; real-time PCR tests on blood samples and nasopharyngeal swabs were used to identify agents.		PCT and MR-proANP helped to identify severe cases.
Naydenova et al. [23]	2016	Gambia	2–59 months	Retrospective Case-control	780 clinically and radiologically confirmed CAP	Only in 84 cases, the aetiology was identified using blood culture.	CRP, Lcn2, Hap and CD163 protein	Aetiology can be determined using three vital signs (RR, HR, and SaO2) and a newly proposed biomarker (lipocalin-2) (81.8% sensitivity and 90.6% specificity). Lcn2 values below 200 ng ml are suggestive of a viral cause. Note: cases with no bacterial growth in blood culture were diagnosed as viral pneumonia. This is not reliable, as blood cultures are not always positive in bacteria CAP. This can lead to diagnostic bias.
						22 bacterial and 62 viral.		
Zhu et al. [24]	2016	China	10 months to 7 years	Prospective cohort	65 based on criteria provided by IDSA and American Thoracic Society	Bacterial *n* = 34;	PCT	Bacterial pneumonia had far greater levels of PCT than non-bacterial. Also, statically significant changes in PCT level were tested before, and after treatment. Therefore, PCT is an important marker.
						Non-bacterial n = 32		
								Note: The type of detection used to isolate the agents are not disclosed in this study.
								Moreover, there is a mismatch between the sample size given in abstract, methods and results.
Engelmann et al. [25]	2015	France	0–16 years	Prospective cohort Multicentre	*n* = 41	Viral clinically* diagnosed *n* = 4.	MxA1	Over 200 ng/ml of MxA has very high sensitivity and specificity in diagnosing a viral infection.
							CRP	
						Viral microbiologically confirmed n = 6		NOTE: Not all cases were microbiologically confirmed as the study design did not accommodate this. The request for confirmation was based on the decision of the treating physician.
						Bacterial clinically* diagnosed n = 16		
						Bacterial microbiologically confirmed n = 6		
						No data *n* = 9		
						*clinical diagnosis included signs and symptoms, and routine laboratory workup such as CRP		
Hoshina et al. [26]	2014	Japan	< 15 years old	Retrospective cohort	*n* = 31	Bacterial *n* = 21	WBC, neutrophil counts, CRP, PCT	PCT was a very useful marker to differentiate bacterial pneumonia. Neutrophil count helped to discriminate bacterial bronchitis.
						Viral *n* = 10		
						Bacterial CAP was confirmed by sputum culture.		
						Viral CAP was confirmed by nasopharyngeal aspirate, sputum or throat swab		
Elemraid et al. [27]	2014 and 2013	United Kingdom	≤16 years	Two prospective aetiological studies	*n* = 241 (in 2002)	2002:	CRP, total WBC, and absolute neutrophil count	CRP and WBC/ neutrophil count can indicate aetiology to a great extent when combined
Elemraid et al. [28]					*n* = 160 (in 2011) based on signs and symptoms and radiographic findings	Viral *n* = 47		
						Bacterial *n* = 58		
						Mixed n = 12		
						2011:		
						Viral n = 50		
						Bacterial *n* = 28		
						Mixed n = 20		
Zhou et al. [29]	2011	China	0.11–2.57 years	Prospective cohort	*n* = 78	Bacteria *n* = 27	CRP, WBC, IgA, IgG, IgM, percent of T, T_c_, T_h_, B, NK, CD23^+^, and CD25^+^cells and degree of expression of HMGB1 mRNA	HMGB1 and WBC can differentiate between bacterial, viral and co-infected cases of bronchial pneumonia
						Viruses *n* = 25		
						Bacteria and viruses *n* = 26		

*REF* Reference, *n*, Sample size, *Lcn2* Lipocalin 2, *SYN4*, Syndecan 4, *CRP* C-reactive protein, *WBC* White blood cell, *IL-10* Interleukin 10, *MR-proADM* Midregional Proadrenomedullin *MR-proANP* Midregional proatrial natriuretic peptide, *PCT* Procalcitonin, *Hap* Haptoglobin, *TNF* Tumor necrosis factor, *TNF-α* Turmos necrosis factor – alpha, *MxA1* Myxoma resistance protein 1, *IgA* Immunoglobulin A, *IgG* Immunoglobulin G, IgM Immunoglobulin M, *T* T-lymphocyte, *T*_*c*_ Cytotoxic Tlymphocyte, *T*_*h*_ Helper T-lymphocyte, *B* B lymphocyte, *NK* Natural Killer, *CD* Cluster of differentiation, *HMGB1 High mobility group box 1* protein, *HPR* Haptoglobin related protein, *ANC* Absolute neutrophil count, *HAdV* Human Adenovirus, *mRNA* Messenger Ribonucleic Acid, *YKL-40* Chitinase-like protein, *BALF* Bronchoalveolar lavage fluids, *HIV* Human immunodeficiency virus

## Results

The search returned 13,408 records up to 1st of July, 2019. After activating filters, we selected 140 articles. After duplicate removal, the title and abstracts were manually sorted and matched according to the inclusion and exclusion criteria. A total of 16 articles were fully reviewed [7, 10–23]. Afterward, details were extracted for each article as described in methods. This information is then summarised in Table 3.

## Discussion

### Standard biomarkers in community-acquired pneumonia

#### C reactive protein (CRP)

A total of eleven studies involved CRP [14, 16, 17, 19, 20, 22, 23, 25–29]. In all the studies analysing CRP as a diagnostic marker, the average CRP level was higher in the bacterial group than viral group [14, 20, 22, 23, 25–29]. In an investigation by Esposito et al. [20] the mean level of CRP was 32.2 mg/L in 74 bacterial CAP cases as compared to 9.4 mg/L from 16 viral CAP cases. Similarly, in a different study by Esposito et al. [22] with larger sample size, the average CRP was 21.3 mg/L in 235 bacterial CAP patients and 8.0 mg/L in 111 viral CAP patients. The study by Bhuiyan et al. [14] showed that CRP concentration was more than 6 times higher in definite bacterial cases than in viral cases. This study also showed that elevation of CRP with the presence or absence of clinical symptoms, such as fever (≥38 °C) or the absence of rhinorrhea differentiates bacterial pneumonia from viral pneumonia better than CRP alone [14]. Although the mean levels of CRP were higher in bacterial CAP, there was overlap between viral and bacterial cases leading to issues in fixing a suitable cut-off point that is both sensitive and specific to differentiate between the two. In the study by Elemraid et al. 25% of viral CAP cases had CRP over 80 mg/L, and nearly 23% of bacterial cases had CRP less than 20 mg/L [27]. It is evident that a low reference point for CRP will diagnose almost all cases of bacterial aetiology but will include a significant number of false-positive cases. For example, with a cut-off value of 10 mg/l, the sensitivity and specificity are 95 and 49% respectively. Doubling the cut-off point to 20 mg/L led to a lower sensitivity of 85% and increased the specificity of 67%. However, a lower threshold value does not guarantee higher sensitivity. In a study by Esposito et al. [20] a cut-off value of 7.4 mg/L only resulted in 64% sensitivity. In a study by Naydenova et al. [23], the diagnostic value of respiratory rate, heart rate, oxygen saturation together with auscultation findings (presence or absence of grunting or crackles) was additionally analysed in association with CRP. The combination of these clinical parameters and CRP slightly improved the predictive power with a sensitivity of 64% and specificity of 88%. In the same study, the author added Lipocalin-2 (Lcn2) to CRP and clinical data which dramatically increased sensitivity to 81.8% and specificity to 90.6%. A significant limitation of almost all the studies was the lack of inclusion of primary care patients. This meant that before hospital admission, many patients might have had exposure to antibiotics which may have altered the level of CRP [27]. Another drawback was that only one study investigated co-infection (viral-bacterial) and concluded that CRP level did not correlate with co-infection [29].

#### Procalcitonin (PCT)

Four studies analysed the diagnostic value of procalcitonin (PCT) [17, 22, 24, 26]. PCT is a precursor to calcitonin produced in the parafollicular cells of the thyroid gland by the transcription of CALC-1 gene. During an infection, CALC-1 gene is activated and upregulated to increase the production of PCT in not only endocrine glands but also many parenchymal tissues [30]. The sudden and marked increase (over 2 ng/ml) in PCT within four to six hours is a key indicator of bacterial infection [31]. It is hypothesized that viruses are not able to increase PCT to such a concentration as certain cytokines expressed during viral infection leads to decreased induction of PCT. This was reflected in the study by Esposito et al. [22] as the mean PCT was 1.1 ng/ml in viral CAP compared to 6.1 ng/ml in bacterial CAP cases. This studyshowed that specificity to identify viral aetiology was higher of PCT compared to CRP. Authors reported that a PCT cut-off value of ≤0.07 ng/ml had the highest combined sensitivity (48.7%) and specificity (81.1%) for viral CAP. Hoshina et al. noted that PCT value higher than 0.2 ng/ml had a sensitivity of 86%, the specificity of 80% to diagnose bacterial pathogen [26].

#### White blood cells (WBC) and neutrophils

Six papers focused on WBC and/or neutrophil count [14, 17, 20, 24, 26, 27]. The total WBC count fluctuates in the paediatric population, especially in the early period of life. Therefore, the reference values differ between the age groups [32]. In general, a value greater than 11 × 10^9/L is considered to be leucocytosis [32]. Research by Elemraid et al. [27] showed that almost 40% of viral pneumonia cases presented with WBC > 15 × 10^9/L. Esposito et al. [20] highlighted that WBC had the lowest positive predictive value compared to PCT and CRP. According to Zhu et al. [24], the percentage of neutrophils compared to a total WBC count was to some extent better at discriminating viral from bacterial infection. According to the literature, passing neutropenia (Neutrophils < 1.5 × 10^9/L) is likely to begin from day three and last until day eight in many viral infections, including RSV, IV and AV [33, 34]. The lack of rise in neutrophil count correlates well with viral causes. In the study by Elemraid et al., 80% of patients with viral pneumonia had neutrophils less than 10 × 10^9/L [27].

### Novel biomarkers

#### Myxoma resistance protein (MxA1)

In recent years new biomarkers have been tested in children. Of this, MxA1 has shown promising results. Compared to other markers, MxA1 protein tends to rise significantly during viral rather than a bacterial infection. Type I or III Interferon (IFN) can activate MxA1 but not type II IFN signaling pathway or the direct interaction of bacteria or viruses [35]. IFN is classified into three groups depending on the similarities in their amino acid sequence. Type I IFN is called alpha, beta, tau, and -omega and are produced in all cells in the body [36]. IFN is also elevated in autoimmune conditions and some hematological cancers. Therefore, the value of IFN induced MxA1 in this population may not be relevant [37].

The study by Engelmann et al. was the largest prospective study analysing the role of MxA1. A cut off value of 200 ng/mL was 96.4% sensitive and 66.7% specific for identifying patients with viral CAP [25]. The author also hypothesized that if a bacterial infection is diagnosed and high levels of MxA1 are detected, this is an indication that bacterial organism preceded a viral cause [25]. This is because MxA1 stays elevated for approximately ten days after a viral insult in comparison to IFN which has a very short half-life [38]. The authors also made a correlation with CRP. Low CRP (< 40 mg/l) and MxA1 > 200 ng/ml is highly indicative of viral aetiology [26]. The study sample size was the biggest drawback of this investigation. Out of 553 children who were enrolled, only 41 had CAP. Moreover, not all cases of CAP were microbiologically confirmed. Therefore, more studies are needed to confirm the diagnostic value of MxA1.

#### Lipocalin 2 (Lcn2)

Lcn2 has been studied in the above data. This protein is stored and released by neutrophils which distorts iron transportation within bacteria. This marker is of a high interest in diagnosing aetiological factor of CAP. Two studies involving Lcn2 were carried out in very different settings which may have contributed to the different results. The study by Esposito et al. [20] concluded that Lcn2 was a poor predictor compared to CRP or WBC [20]. While Naydenova et al. [23] found the use of Lcn2 to help discriminate bacterial and viral pneumonia. The latter study was set in a developing nation amongst children with malaria, which is known to affect the concentration of Lcn2 [39]. According to Huang et al. [39], a cut-off value of more than 130 ng/ml strongly correlates with bacterial aetiology (sensitivity 83.67% and specificity 85.71%). Also, Lcn2 of more than 160 ng/ml is highly indicative of a positive isolate from a blood culture [39].

#### High mobility group box one protein (HMGB1)

HMGB1 is a protein which binds to DNA and causes the transcription of several inflammatory markers. Furthermore, it has some extracellular roles such as promoting migration and enhancing the production of pro-inflammatory markers and cytokines such as Interleukin 6 (IL-6), Tumour necrosis factor (TNF) or Interferon gamma (IFN-γ). This protein is elevated during CAP, sepsis and viral-bacterial co-infections, especially bacterial and Influenza virus co-infection [40]. A study by Zhou et al. [29] evaluated changes in the expression of HMGB1 gene in peripheral monocytes as opposed to measuring the concentration of HMGB1 in the serum. By using the PCR technique, gene expression was quantified by comparing HMGB1 proteins density to an *18S ribosomal ribonucleotide acid*. It was found that co-infection (virus and bacteria) can be concluded when HMGB1 expression is greater than 1.0256. Furthermore, in this study HMGB1 expression < 1.0256 and a WBC > 13 × 10^9^/L had 92.3% positive predictive value for single bacterial pneumonia [29]. Therefore, HMGB1 seems to be a good marker. However, the need to isolate specific blood cells (monocytes) and to adopt PCR makes this method prolonged and expensive. Moreover, measurement of RNA may not necessarily correlate with functional serum HMGB1 protein.

#### Other markers

Several new markers, such as Syndecan 4 (SYN4), were explored with poor reliability. Results from the study by Esposito et al. revealed that SYN4 had an AUC on ROC of only 0.54 (95% CI 0.40–0.69) compared to 0.67 (95% CI 0.53–0.80) for CRP [20]. Proteins that can affect the cardiovascular system: Midregional Proadrenomedullin and Midregional pro-atrial natriuretic peptide were found to be not useful in predicting aetiology; instead, these were indicative of CAP severity [22]. Few microRNAs have been recently reported as a biomarker for several diseases. Study by Huang et al. [15] found that miR-450a-5p/miR-103a-3p and miR-103b/miR-98-5p could be considered as potential diagnostic biomarkers for adenovirus infection-associated pneumonia.

It is willing that another recently discovered biomarker chitinase-like protein (YKL-40) could also be used to differentiate viral and bacterial pneumonia. This protein is involved in airway imflammation and potential of this marker is under observation. The recent study by Yang et al. [16] showed that levels of YKL-40 in the bronchoalveolar lavage fluids specimens compared with serum levels of patients with bacterial pneumonia were significantly higher than with viral pneumonia [16]. This study also showed that YKL-40 reductions in serum levels on day 5 after receiving therapy is a possible prognostic biomarker for children with viral pneumonia [16]. The results of this study let us believe that YKL-40 has potential value in the differentiating viral and bacterial pneumonia.

### Marker combinations

Valim et al. [21], evaluated 56 plasma proteins in training set, validation set, and healthy controls in order to distinguish bacterial, viral, and malaria in children presenting with clinical signs and symptoms of pneumonia. The result of the study found that combining haptoglobin (Hap), tissue inhibitor of metalloproteinases-1, Interleukin 19 (IL-19) or TNF receptor 2 resulted in a sensitivity of 96% and specificity of 86% in bacterial diagnosis CAP.

Meanwhile, Elemraid et al. [27] advocated for a rather simple combination of age, CRP, and WBC together with neutrophils count. This discriminatory model had 91.4% positive predictive value and 71.2% negative predictive value for bacterial CAP in children under 16.

Utilizing an extended number of markers with clinical signs does not improve the sensitivity or specificity according to Naydenova et al. [23]. Sensitivity and specificity when combining respiratory rate, heart rate, and oxygen saturation with Lnc2 was 82 and 91%, respectively. Adding CRP or Hap to this did not improve sensitivity or specificity.

## Conclusions

It is very challenging to accurately predict bacterial or viral pneumonia on clinical, radiological as well as on laboratory grounds. As far as the clinical picture is concerned, a child under the age of five, who is sub-febrile with lung field changes and is wheezing is most likely to present with a viral CAP.

With serum markers, the differences in cut-off values are related to the differences in detection methods, analyses, the desired specificity and sensitivity and the presence of mixed infection. From the results, almost all markers had a higher value in bacterial pneumonia. The only marker increased in viral pneumonia and not in bacterial pneumonia was MxA1. This is a promising development, and more studies need to be instituted, and if results are consistent, it may be an essential marker to rule in or out the viral infections. Furthermore, co-infection was a constant dilemma in many studies. Although HMBG1 expression was vital in proving mixed infection, the need for PCR makes this test non-viable in clinical settings. Therefore, similar studies are needed to be conducted to measure the HMBG1 protein concentration in serum rather than the gene expression (Table 4).

**Table 4:** A summary of the markers with the cut-off values

	[REF]	Bacterial	Viral	Bacterial and Viral	Specificity	Sensitivity
CRP	[14]	> 72 mg/L			84.0%	75.0%
	[20]	> 7.4 mg/L			69.4%	64.%
	[20]		<5.2 mg/L		64.5%	75.0%
	[22]	> 7.98 mg/L			53.8%	63.5%
	[22]		< 7.5 mg/L		46.3%	88.2%
	[27]	> 80 mg/L			90.0%	68.1%
PCT	[22]	> 0.188 ng/ml			65.1%	67.4%
	[22]		< 0.07 ng/ml		81.1%	48.7%
	[26]	> 0.2 ng/ml			80%	86%,
	[20]	≥13.500(*S. pneumoniae)*			68.6%	63.8%
	[20]	≥10.300			38.9%	74.0%
	[20]		≤19.710		28.0%	93.7%
WBC	[22]	> 12,870			61.3%	41.6%
	[22]		< 1570		33.1%	78.4%
Neutrophils	[22]	> 61.0%			53.8%	63.5%
	[22]		< 60.8%		60.1%	56.9%
Lcn2	[23]		< 200 ng/ml			
	[20]	≥1633 ng/ml (*S. pneumoniae)*			62.7%	71.2%
		≥1633 ng/m l			50.0%	58.1%
			≥896 ng/ml		28.7%	87.5%
MR-proADM	[22]	> 0.32 nmol/L			35.7%	78.0%
	[22]		< 0.31 nmol/L		73.4%	35.8%
MxA1	[25]		> 200 ng/mL		66.7%	96.4%
HMGB1(expression)	[29]			> 1.0256	67.9%	88%
YKL-40 (BALF)	[16]	26.45 ± 3.65 ng/ml	34.87 ± 5.42 ng/ml	33.63 ± 2.50 ng/ml		
YKL-40 (serum)	[16]	18.48 ± 4.63 ng/ml	19.38 ± 3.34 ng/ml	19.32 ± 2.87 ng/ml		
MxA1 + CRP	[25]			> 200 ng/ml+ > 40 mg/L		

*REF* Reference, *CRP* C-reactive protein, *PCT* Procalcitonin, *WBC* White blood cell, *Lcn2* Lipocalin, *MR-proADM* Midregional Proadrenomedullin, *MxA1* Myxoma resistance protein, *HMGB1 High mobility group box 1* protein; *ng* nanograms, *nmol* nanomolar, *mL* milliliter, *L* Liter, *mg* milligram, *BALF* Bronchoalveolar lavage fluids

One approach is to make use of more than one marker and combine with clinical signs and symptoms. Lnc2, when combined with clinical features was 82% sensitive and 91% specific for bacterial CAP [23]. Lnc2 performed better than CRP, and therefore a solution is to include Lnc2 during laboratory work-up. When higher sensitivity and specificity is needed, combining Haptoglobin (Hap), tissue inhibitor of metalloproteinases-1, Interleukin 19 (IL-19) or TNF receptor 2 could be a solution. However, this may not be cost-effective in many clinical settings.

## Practical recommendations

The optimal cut-off values for different markers and more studies are needed to provide more accurate results and associate it with patients or within the context of the clinical situation, and whether the aim is to diagnose bacterial CAP or viral CAP. Adding Lnc2 to clinical context together with CRP should be considered for better predictive power. Also, consider the combination Hap, tissue inhibitor of metalloproteinases-1, IL-19 or TNF receptor 2 if resources are available.

## Data Availability

The data that support the findings of this study are available within the article and via the referenced articles (depending on institution agreement referenced article might not be free of charge or open-access).

## Abbreviations

ANC: Absolute neutrophil count
AUC: Area under the curve
AV: Adenovirus
B: B Lymphocyte
BALF: Bronchoalveolar lavage fluids
CAP: Community-acquired pneumonia
CD: Cluster of differentiation
CI: Confidence interval
CRP: C-reactive protein
DNA: Deocyribonucleic acid
HadV: Human adenovirus
Hap: Haptoglobin
HIV: Human immunodeficiency virus
HMGB1: High mobility group box 1 protein
IFN: Interferon
IFN-γ: Interferon-gamma
IgA: Immunoglobulin A
IgG: Immunoglobulin G
IgM: Immunoglobulin M
IL:10: Interleukin − 10
IL:19: Interleukin-19
IL:6: Interleukin-6
IV: Influenza Virus
L: Liter
Lcn2: Lipocalin-2
mg: Milligram
mL: Milliliter
mRNA: Messenger Ribonucleic acid
MR-proADM: Midregional proadrenomedullin
MR-proANP: Midregional proatrial natriuretic peptide
MxA1: Myxoma resistance protein 1
n: Sample size
N/L, NLR: Neutrophil-lymphocyte ratio
ng: Nanograms
NK: Natural Killer
nmol: Nanomolar
PCT: Procalcitonin
REF: Reference
RNA: Ribonucleic acid
ROC: Receiver operating characteristic curve
RSV: Respiratory syncytial virus
SYN4: Syndecan-4
Tc: Cytotoxic T- lymphocyte
Th: Hepler T- lymphocyte
TNF: Tumour necrosis factor
TNF-α: Tumour necrosis factor-alpha
WBC: White blood cells
YKL-40: Chitinase-like protein
